# Longitudinal Effects of Physical Inactivity and Obesity on Gait Speed in Older Adults with Frequent Knee Pain: Data from the Osteoarthritis Initiative

**DOI:** 10.3390/ijerph120201849

**Published:** 2015-02-05

**Authors:** Saad M. Bindawas, Vishal Vennu

**Affiliations:** Physical Therapy Program, College of Applied Medical Sciences, King Saud University, P.O. Box 10219, Riyadh 11433, Saudi Arabia; E-Mail: vvennu@ksu.edu.sa

**Keywords:** physical inactivity, obesity, gait speed, knee pain

## Abstract

Physical inactivity (PI) and obesity are risk factors for many health conditions, including knee pain (KP). The purpose of the present study was to examine the 6-year effects of PI and obesity on gait speed (GS) among older adults with frequent KP. This prospective cohort study used data from the Osteoarthritis Initiative (OAI). At baseline, we studied 1788 adults aged 45 to 79 years old. We grouped the participants into four categories according to baseline scores on the Physical Activity Scale for the Elderly (PASE) and body mass index (BMI). GS was measured using the 20-m timed walk test. Frequent KP was assessed with a self-report questionnaire, and obesity was assessed by BMI (30 kg/m^2^ or greater). General linear mixed models were conducted using data collected at baseline and 12, 24, 36, 48, and 72 months. After adjusting for all covariates, lower levels of physical activity and obesity were associated with a decrease in GS (β = −0.095, SE = 0.011, *p* < 0.0001). Our results suggest that both PI and obesity are associated with decreased GS over time in older adults with frequent KP.

## 1. Introduction

Frequent knee pain (KP), the main symptom of knee osteoarthritis (OA), may limit physical activity (PA), most evidently as reflected by a person’s gait speed (GS) [1]. The National Health Interview Survey (NHIS) [2] and the Canadian National Population Health Survey (CNHS) [3] reported that KP is commonly viewed as a barrier to PA for older adults, especially those with knee OA. Low levels of PA increase in prevalence with aging and are associated with negative health outcomes [4], including chronic comorbidity, musculoskeletal disorders, and reduced quality of life [5].

Globally, the World Health Organization (WHO) has identified physical inactivity (PI) as the fourth leading risk factor for mortality [6]. In the United States (U.S.), less than half (48%) of all adults meet the 2008 PA guidelines; in particular, men (52%) are more likely than women (43%)to fail to meet the guidelines [7]. In the U.S., 23% of the population is physically inactive, especially Americans living in the South [8]. In addition, insufficient PA places a heavy financial burden on the economy worldwide. For example, the estimated financial burden for PI in the U.S. was $93.32 billion in 2008 [9]. Recent studies suggest that PA may modify genetic susceptibility to obesity; physically inactive people were more likely to have a high genetic burden than active individuals [10].

The prevalence of obesity has increased in many developed countries since the 1970s [11]. In the U.S., the prevalence of obesity is 29.4% in males and 14.5% in females, and more than 78.6 million adults (one-third of the population) are obese [12]. Recent data have shown that obesity is associated with decreased GS and could predict disability [13]. Researchers have suggested that slower GS develops because of an excess of the body weight load on the knee joints while walking in adults with frequent KP compared with normal individuals [14]. Because older age along with frequent KP have been associated with slower GS [15], it is reasonable to suggest that the effects of obesity on GS are likely greater in older adults with frequent KP [11]. In addition, excess weight places a heavy financial burden on the economy worldwide. For example, the estimated financial burden linked to excess weight in the U.S. was $94.33 billion in 2008 [9].

GS is an important predictor of major health outcomes in older adults, including hospitalization [16], limited activities of daily living (ADL) [17], and impaired health and functioning [18]. Thus, GS has been strongly associated with functional capacity and is a vital sign in older adults [13]. Previous studies have reported that GS decreased at a rate of 1%–4% per year in older adults aged 65 years or older [19]. However, the impact of both PI and obesity on GS in older adults with frequent KP is not known. The present study provided information on the specific risk factors (*i.e.*, PI, obesity or a combination of both) and how they are associated with a decrease over time in GS in older adults with frequent KP. This information is important for clinicians because they typically focus mainly on pain management to improve GS in older adults with frequent KP [20].

To our knowledge, no previous longitudinal studies have examined the impact of both PI and obesity on GS in older adults with frequent KP. Therefore, the purpose of the present study was to examine the 6-year effects of PI and obesity on GS in those with frequent KP. We hypothesized that both conditions would be associated with decreased GS over time, among older adults with frequent KP, compared to those who are physically active and not obese.

## 2. Methods

### 2.1. Study Design

This is a six-year longitudinal cohort study using data from the Osteoarthritis Initiative (OAI).

### 2.2. Setting

The OAI is an ongoing publicly and privately funded, multi-center, longitudinal cohort study (accessible to the public at http://www.oai.ucsf.edu) [21]. Participants aged 45–79 years at baseline were recruited from four clinical sites in the U.S.—Baltimore, MD; Pittsburgh, PA; Pawtucket, RI; and Columbus, OH—between February 2004 and May 2006.

### 2.3. Participants

The sample for the current study consists of 1788 older adults from the OAI baseline. The participants were grouped into four categories according to PA level and body mass index as follows: a high-PA and non-obese (reference) group, a low-PA and non-obese group, a high-PA and obese group, and a low-PA and obese group.

**Figure 1:**
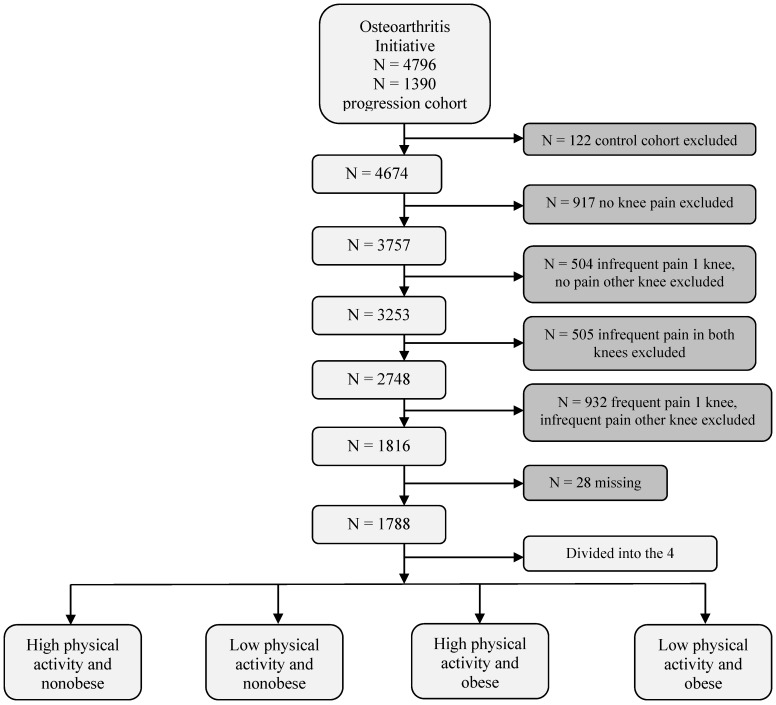
Flow diagram of selection and classification of participants enrolled in the Osteoarthritis Initiative study that was included in the present study.Note: Osteoarthritis Initiative online database provided by the coordinating center, University of California, San Francisco (https://oai.epi-ucsf.org/datarelease/).

Participants were included if they were aged 45–79 years, regardless of sex or ethnicity (Figure 1). We excluded participants who had no KP (*N* = 917), infrequent pain in one knee and no pain the other knee (*N* = 504), infrequent pain in both knees (*N* = 505), or frequent pain in one knee and infrequent pain in the other knee (*N* = 932), as well as participants who did not know, were not sure, or refused to answer (<2% of the 1,788 respondents) [21] from the analyses (Figure 1).

Each participant was instructed about the objectives and research procedures, and all participants provided signed informed consent. The OAI study protocol was approved by the institutional review boards at the OAI Coordinating Center at the University of California, San Francisco.

### 2.4. Measurements

**Frequent KP** was defined as a “yes” answer to both of the two following self-reported questions: (1) “During the past 12 months, have you had pain, aching or stiffness in or around your (right/left) knee?” and (2) “During the past 12 months, have you had pain, aching or stiffness in or around your (right/left) knee on most days for at least 1 month?” Participants were classified as not having frequent KP if they answered “no” to both questions or as having infrequent KP if they answered “yes” to the first question but “no” to the second. Similar questions have been used in the Baltimore Longitudinal Study of Aging [22] and other population-based surveys [23].

Physical activity level was assessed using the Physical Activity Scale for the Elderly (PASE) [24], an established 12-item questionnaire for measuring self-reported PA in three life domains (leisure, household and occupational activities) among older adults. A series of twelve questions is asked about PA levels over the previous seven days. The resulting score is rounded to the nearest integer, with a theoretical range of 0 to over 400. Higher scores indicate a higher level of PA. If the response to any of the required 12 questions or subquestions is missing, then the calculated score is set to a special missing value. The median PASE score for all participants was 151.5, calculated as previously described [25]. On the basis of PA level, participants were divided into the two groups: high-PA (PASE score: 150–400) and low-PA (PASE score: 0–149). Similar PA levels were used in the Stehling *et al*., study [25].

**Weight** in kilograms (kg) was measured twice at the clinic visit. The two values were averaged. If one value was missing, the remaining value was used. **Height** in millimeter (mm) was measured twice then compared. If the difference was more than 1 mm, it was measured twice more. If height was measured four times, the last two were averaged. If there were three values (*i.e.*, one of the first or second pair was missing), all three values were averaged. If only one value was recorded, it was used. A check was run to ensure that the larger value did not exceed the smallest by more than 20% in both the weight and height measures. The body mass index (BMI) was calculated as weight divided by the square of height in meters (m). On the basis of BMI, participants were divided into two groups: non-obese (BMI < 30) and obese (BMI ≥ 30), as suggested by the WHO [26].

**Gait speed (meter/second)** was assessed by a timed 20-m walk test using a stopwatch at baseline and in the same participants over the 6-year follow-up. The 20-m walk pace was calculated as an average pace over two trials during the each clinic visit. If one value was missing, the remaining value was used. The majority of epidemiological studies has used the timed 20-m walk test; it is a standard outcome measure for osteoarthritis [27]. The participants walked a comfortable, self-directed pace in a corridor between two cones spaced 20-m apart, and then they returned to sit in a chair at the starting cone. The time to complete the first 20-m walk was recorded in meters per second. GS was computed as the distance to complete the test divided by the time, similar to other epidemiological studies [28]. The participants wore their usual footwear and used walking aids/devices as needed.

The following sociodemographic characteristics were included in the analysis: age (in years), race (white *vs*. “other” (black, Asian, other non-white)), marital status (married *vs*. unmarried/widows/divorce), education level (high school graduates or less *vs*. college graduation), and income in U.S. dollars (<$50,000 *vs*. ≥$50, 000). Health status encompassed the number of self reported chronic conditions as comorbidities, depression, and physical and mental composite scales of the Medical Outcomes Study 12-Items Short Form (SF-12). The Charlson comorbidity index was used to measure comorbidity [29]. As an indicator of highly depressive symptoms, we used a score ≥ 16 on the Center for Epidemiological Studies Depression Scale [30]. The SF-12 was used to assess the participants’ physical and mental health [31].

### 2.5. Statistical Analysis

Patient characteristics and outcomes were examined using univariate descriptive statistics. A one-way analysis of variance (ANOVA) with Tukey *post hoc* test and chi-squared tests were used to assess univariate differences between continuous and categorical variables, respectively. Subgroups were used as independent variables for one-way ANOVAs, and sociodemographic (age, sex, race, marital status, education, and income) and health (comorbidity, depression symptoms, physical composite scale (PCS) and mental composite scale (MCS)) factors were used as independent variables for chi-squared tests. General linear mixed model using the MIXED procedure in SAS (SAS Institute, Inc., Cary, NC, USA) was used to predict the longitudinal impact of low-PA, obesity or both conditions on GS in older adults with frequent KP at baseline and over the 6-year follow-up period. Based on PA levels and obesity status at baseline, the sample were categorized into four groups and changes in GS were observed over time. We used an unstructured covariance matrix to determine the associations among PI, obesity and GS over time. A mixed model enabled us to make inferences regarding the follow-up by modeling and estimating the components. Furthermore, this approach made better use of incomplete data, such as data for participants who dropped out or missed scheduled follow-up visits [32].

Six mixed models were constructed to test the independent relationship and both conditions between PA levels and obesity status at baseline and over a 6-year period. Model 1 included time, groups. In Model 2, age, sex, race, marital status, education, and income were added to the variables in Model 1. In Model 3, depression, PCS, and MCS were added to the variables in Model 2. All Models used an interaction term between groups and time. Analyses were performed using the SAS system for Windows version 9.2 (SAS Institute, Inc.). To measure the effect size, we calculated the Cohen’s f^2^ [33] as described by Selya *et al*. [34].

## 3. Results

Of the total of 1788 participants the average mean (SD) age at baseline was 60.2 (8.7) years old, with a range of 45 to 79 years. The vast majority of the participants were white (70%), female (58%), and married (62%); had an education level of high school graduate (57%); reported no depressive symptoms. Participants in a low PA and obese group were obese had more mean comorbidity, lower GS, PCS, and PASE scores respectively (Table 1).

**Table 1:** Characteristics of the study participants (1788).

Characteristics, N (%)	ALL *N* = 1788	High Physical Activity and Non-obese *N* = 537	Low Physical Activity and Non-obese *N* = 507	High Physical Activity and Obese *N* = 340	Low Physical Activity and Obese *N* = 404	*p Value*
**Females**	1,034 (57.83)	283 (27.37)	294(28.43)	188 (18.18)	269 (26.02)	*0.0002*
**White or Caucasian**	1,254 (70.13)	436 (34.77)	383 (30.54)	213 (16.99)	222 (17.70)	*<0.0001*
**High school graduates or less**	474 (57.18)	161 (33.97)	138 (29.11)	91 (19.20)	84 (17.72)	*<0.0001*
**Married**	1,110 (62.08)	360 (32.43)	321 (28.92)	205 (18.47)	224 (20.18)	*0.003*
**Annual income (<$50,000)**	732 (40.94)	179 (24.45)	206 (28.14)	120 (16.39)	227 (31.01)	*<0.0001*
**Depressive symptoms (yes)**	245 (13.70)	37 (15.10)	88 (35.92)	49 (20.00)	71 (28.98)	*<0.0001*
**Characteristics, mean ± SD**										
**Age (yrs)**	60.2 ± 8.7	58.9 ± 8.8	63.6 ± 9.4	56.5 ± 7.5	61.6 ± 8.9	*<0.0001*
**Comorbidity**	0.47 ± 0.9	0.31± 0.8	0.44 ± 0.8	0.45 ± 0.8	0.70 ± 1.2	*<0.0001*
**Gait Speed (meter/second)**	1.27 ± 0.2	1.38 ± 0.2	1.27 ± 0.2	1.28 ± 0.2	1.17 ± 0.2	*<0.0001*
**BMI (kilogram/ meter square)**	30.0 ± 2.9	25.9 ± 2.5	26.0 ± 2.7	33.9 ± 3.2	34.0 ± 3.5	*<0.0001*
**Physical Composite Scale (PCS)**	45.4 ± 9.7	49.6 ± 8.4	45.5 ± 10.0	45.4 ± 9.2	41.0 ± 11.2	*<0.0001*
**Mental Composite Scale (MCS)**	52.7 ± 9.1	53.8 ± 7.6	52.3 ± 10.0	52.2 ± 8.9	52.6 ± 10.1	*0.021*
**Physical Activity Scale for the Elderly (PASE)**	161.0 ± 49.2	227.8 ± 59.5	97.0 ± 35.2	232.9 ± 65.0	86.6 ± 37.1	*<0.0001*

yrs = years; BMI = body mass index; $ = USD.

Table 2 shows the general linear mixed models estimates of gait speed as a function of physical activity levels (low *vs*. high) and obesity status (obese *vs*. not obese) over the 6-year period. The adjusted rate of decreased in GS was 0.0014 m/s per year in both individual interactions of PI and obese (Table 2). Individually, PI and obesity were significantly associated with lower GS in all models. After adjusting for all covariates, PI (β = −0.035, standard error (SE) = 0.009) and obesity (β = −0.078, SE = 0.009) were significantly independently associated with decreased GS with a small effect size. The separate interaction between low PA, obesity and time were not statistically significant in all models, indicating unchanged slop. It means that, every year, the average GS of older adults observed decreased by 0.0014 m/s.

Table 3 shows General Linear Mixed Models Estimates for Gait Speed in four groups based on high or low Physical Activity (PA) levels and obesity status over 6 years in older adults with frequent knee pain. In Model 1 (unadjusted), compared with the reference group, the low-PA and non-obese group (β = −0.089, SE = 0.011), the high-PA and obese group (β = −0.093, SE = 0.012), and the low-PA and obese group (β = −0.18, SE = 0.012) were significantly associated with decreased GS with a small effect size. In Model 2, after adjusting for sociodemographic variables (age, sex, race, marital status, education, and income), the low-PA and non-obese group (β = −0.039, SE = 0.0099), high-PA and obese group (β = −0.078, SE = 0.011), and the low-PA and obese group (β = −0.11, SE = 0.011) were significantly associated with decreased GS with a small effect size compared with the reference group. In Model 3, after controlling for depression, PCS, and MCS, together with the variables in Model 2, the low-PA and non-obese group (β = −0.037, SE = 0.0099), the high-PA and obese group (β = −0.061, SE = 0.011), and the low-PA and obese group (β = −0.095, SE = 0.011) were significantly associated with decreased GS with a small effect size. The interaction term between groups (high PA and obese; low PA and non-obese; and low PA and obese) and time of follow-up (GS score over 6 years) was not statistically significant in all models, indicating unchanged slop. It means that, every year, the average GS of older adults observed decreased by 0.0014 m/s.

**Table 2:** General linear mixed models estimate for gait speed as a function of physical activity (PA) levels and obesity status over 6 years in older adults with frequent knee pain (*N* = 1788).

Predictor Variable	Model 1		Model 2		Model 3	
	β	SE	β	SE	β	SE
Intercept	1.37 *	0.0080	1.91 *	0.033	1.49 *	0.039
Time	−0.00025	0.0008	−0.0008	0.0008	−0.0014	0.0008
Low physical activity *vs*. high physical activity	−0.089 *	0.011	**−0.038** **	0.010	−0.035 **	0.009
Obese *vs*. non-obese	−0.14 *	0.010	−0.097 *	0.0095	−0.078 *	0.009
**High physical activity and non-obese by time**						
Low physical activity	−0.0010	0.0012	−0.0012	0.0011	−0.0003	0.0011
Obese	−0.00076	0.0010	−0.0008	0.0010	−0.0002	0.0010

β = estimate; SE = standard error. Model 1 included time. Model 2 included the Model 1 plus age, sex, race, education, marital status, and income. Model 3 included the Model 2 plus center for epidemiological scale for depression, physical and mental composite scale of the SF-12. * *p* < 0.0001; ** *p* < 0.01.

**Table 3:** General linear mixed models estimate for gait speed in four groups based on physical activity (PA) levels and obesity status over 6 years in older adults with frequent knee pain (*N* = 1788).

Predictor variables	Model 1		Model 2		Model 3	
	β	SE	β	SE	β	SE
**Four groups based on high or low PA levels and obesity status**						
Intercept	1.37 *	0.0080	1.89 *	0.033	1.48 *	0.038
Time	−0.0003	0.0008	−0.0007	0.0008	−0.0014	0.0008
1. High physical activity and non-obese	**Reference**	**Reference**	**Reference**			
2. Low physical activity and non-obese	−0.089 *	0.011	−0.039 **	0.0099	−0.037 **	0.0099
3. High physical activity and obese	−0.093 *	0.012	−0.078 *	0.011	−0.061 *	0.011
4. Low physical activity and obese	−0.18 *	0.012	−0.11 *	0.011	−0.095 *	0.011
**Four groups by time**							
1. High physical activity and non-obese	**Reference**	**Reference**	**Reference**	**Reference**	**Reference**	**Reference**
2. Low physical activity and non-obese	−0.001	0.0012	−0.0012	−0.001	0.0012	−0.0012
3. High physical activity and obese	−0.0028	0.0013	−0.00026	−0.0028	0.0013	−0.00026
4. Low physical activity and obese	−0.0016	0.0012	−0.0012	−0.0016	0.0012	−0.0012

β = estimate; SE = standard error. Model 1 included time. Model 2 included the Model 1 plus age, sex, race, education, marital status, and income. Model 3 included the Model 2 plus center for epidemiological scale for depression, physical and mental composite scale of the SF-12. * *p* < 0.0001; ** *p* < 0.01.

**Figure 2:**
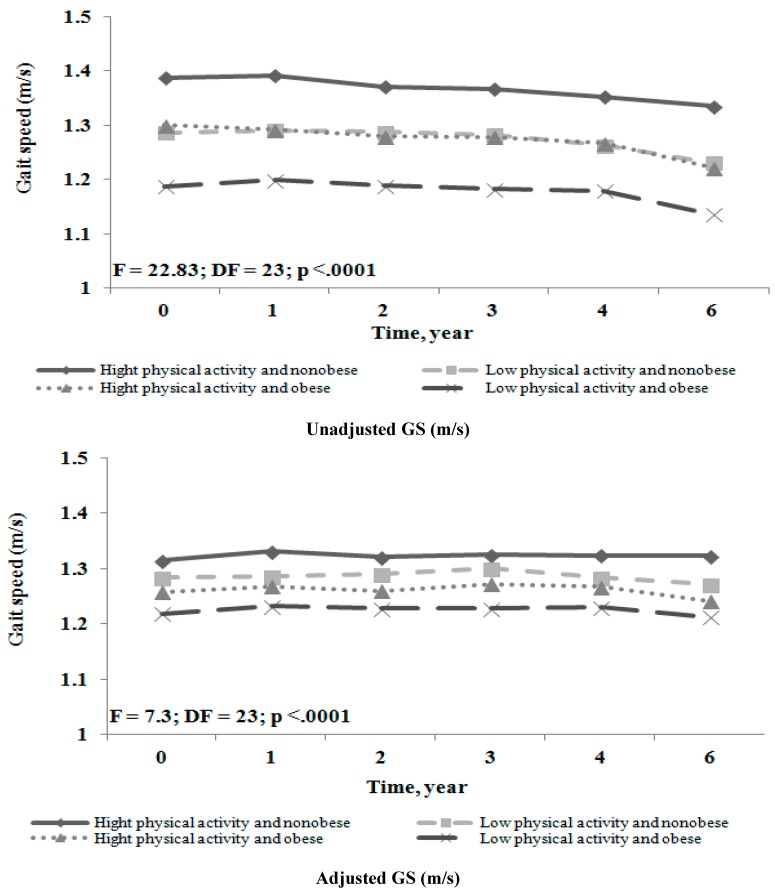
Unadjusted and adjusted GS over 6-year period in older adults with frequent knee pain (unadjusted GS (m/s) —older adult’s statistically significant, *p* < 0.0001; adjusted GS (m/s)—older adult’s statistically significant, *p* < 0.0001).

Figure 2 shows the unadjusted and adjusted GS over the 6-year period, according to the both high or low PA levels and obesity status at baseline. Participant with both conditions, low PA and obese had slower GS followed by low PA and non-obese and high PA and obese than participants in the reference group over the entire follow-up period. Moreover, participants in all groups had stable gait speed over the six years.

## 4. Discussion

To our knowledge, this is the first study to explore the longitudinal effects of PI and obesity on GS among older adults with frequent KP, aged ≥ 45 years and older. The findings support the hypothesis that both conditions would be associated with decreased GS over time, among older adults with frequent KP, compared to those who are physically active and not obese.

In our study, the overall GS estimate for both conditions was decreased by 0.095 m/s, after adjusting for all covariates. These estimates are quite similar to what is regarded as normal for community-dwelling older adults. There is a disagreement between the studies about GS norms, especially between clinical and observational studies. In community-dwelling older adults usual GS mean values very substantial. For example, from 0.56 m/s in a Hispanic American population to 1.19 m/s in a population of White Americans [35]. Bohannon *et al*., presents reference values for comfortable GS for women (1.16 m/s) and men (1.28 m/s) age 50–79 years [36].

Our findings are similar to findings in older population showing that individually PI and obesity had an effect on GS in older adults than in normal populations reduced GS [37,38]. In the current study, we found that in both unadjusted and adjusted models with sociodemographic and health covariates, the presence of both conditions had a greater impact on GS over the 6-year follow-up period compared with participants who had high PA and non-obese. The presence of obesity alone had a greater impact on GS among older adults with frequent KP compared with PI alone. Thus, it appeared that GS was affected more in obese adults with frequent KP due to the continuous pain and excess body weight load on the knee joint while walking compared with normal individuals [14].

These results are consistent with a previous study of Anderson *et al*. using data from a cohort of 8000 U.S. health plan members aged ≥ 40 years, found that higher total health care charges were associated with PI and obesity [39]. Thus, having both conditions causes a heavy financial burden on the economy as well as negative health outcomes, including lower GS among older adults with frequent KP in the U.S. population [40]. Dunlop *et al*. in a study of 2589 people with knee OA aged 45–79 years at baseline, showed a consistent graded relationship between PA level and GS. Their findings indicated that high levels of PA were significantly associated with a higher GS ever after controlling for sociodemographic and health covariates [27]. The results of a prospective cohort study by Mansournia *et al*. suggested that the PA had either no effect or a very small effect on GS and did not affect KP in adults with knee OA [28].

Older adults with frequent KP report lower level of PA and obese than older adults with no KP [41]. The mean PASE score for low PA and non-obese as well as low PA and obese groups were 97.0 ± 35.2 and 86.6 ± 37.1 respectively, which is lower than the average score of 118.9 in older adults in a validity study for the PASE [42] and lower than the median sample score of 151.5 of the current study samples. The mean age of groups with high physical activity was about five years lesser than in groups with low physical activity, thus gait speed should be influenced not only by activity level, but also by mean age of group [4]. Himann *et al*., reported that, 62 years coincided with a decline in GS. Before 62 years, there was a 1% to 2% per year decline in normal GS [19].

Our study strengths were as follows, using a prospective cohort design for, a large sample size. Also, gait speed was measured by using the 20-m timed walk test, which is widely used to assess GS in older adults and has been validated in similar participants [28]. Finally, to our knowledge this is the first longitudinal study to report the effect of both PI and obesity on GS in adults with frequent KP.

Our study limitations are that the PA and KP measures were based on self-report, and PA and BMI were available only at baseline, which makes it difficult to determine the changes of KP and the participants PA levels over time. However, the validity of PASE has been established in older adults with KP as a reasonable tool to discriminate those who are more physically active from those who are not [43]. Second, using older participants makes follow-up studies inherently biased, as unhealthy participants are less likely to be included over time. Therefore, Mixed Models were used in this study, which allows the use dependent effect [44,45]. In addition, these findings apply to older adults with frequent KP and cannot be generalized to the normal population.

Physical activity and obesity are two important indicators for individual and population health. A better understanding of the impact of physical inactivity and obesity on function as measured by GS is necessary to develop effective prevention and treatment programs, particularly for older adults with knee pain [46]. For example, controlling lifestyle-related behaviors such as the amount of physical activity, consuming a balanced diet, and maintaining a healthy weight, my prevent, delay, or improve healthy life including independence in functional performance [47,48].

## 5. Conclusions

We found that older adults with frequent knee pain who had both physical inactivity and obesity had a slower GS over the 6-year follow-up period compared with a participant who had a high PA and non-obese. PI and obesity were significantly associated with lower GS even after adjusting for sociodemographic and health variables. Obesity alone was more strongly associated with GS then was PI alone, even after adjusting for all covariates. Overall, the amount of decrease in GS in participants with frequent KP was larger among those with both PI and obesity than in high-PA and non-obese individuals. Additional studies are needed to verify the effects of these conditions on GS in adults with other musculoskeletal conditions.
